# Airway management during left-sided gastrobronchial fistula repair after esophagectomy for esophageal carcinoma: A case report

**DOI:** 10.1097/MD.0000000000027133

**Published:** 2021-09-03

**Authors:** Sih-Yu Wang, Wei-Chin Yuan, En-Bo Wu

**Affiliations:** Department of Anesthesiology, China Medical University Hospital, China Medical University, Taichung, Taiwan.

**Keywords:** airway management, bispectral index, gastrobronchial fistula, hypoxic pulmonary vasoconstriction, total intravenous anesthesia

## Abstract

**Rationale:**

Gastrobronchial fistula (GBF) is a rare but life-threatening complication of esophagectomy with gastric conduit reconstruction, and airway management during fistula repair is challenging. Here, we describe airway management in a patient undergoing left-sided GBF repair using video-assisted thoracoscopic surgery.

**Patient concerns:**

A 63-year-old man diagnosed with esophageal carcinoma underwent esophagectomy with reconstruction by gastric pull-up and tabularization of the gastric conduit. Subsequently, about 8 weeks later, the patient presented with repeated pneumonia and a 1-week history of cough with significant sputum, dysphagia, and repeated fever.

**Diagnosis:**

GBF, a rare postoperative complication, was located on the left main bronchus at 2 cm below the carina and was diagnosed based on findings from gastroscopy, flexible bronchoscopy, and thoracic computed tomography scan with contrast.

**Interventions:**

We performed left-sided one-lung ventilation (OLV) under total intravenous anesthesia instead of inhalational anesthetics. The left-sided OLV, with positive end-expiratory pressure (PEEP) and nasogastric tube decompression, generated positive pressure across the fistula. It prevented backflow into the left main bronchus. Total intravenous anesthesia preserved hypoxic pulmonary vasoconstriction and prevented adverse effects associated with inhalational anesthetics. A right-sided, double-lumen endotracheal tube was inserted after anesthesia induction, and surgical repair was performed through a right-sided video-assisted thoracoscopic surgery.

**Outcomes:**

Intraoperative hemodynamics remained relatively stable, except for brief tachycardia at 113 beats/min. Arterial blood gas analysis revealed pH 7.17 and PaO_2_ 89.1 mmHg upon 100% oxygenation, along with hypercapnia (PaCO_2_ 77.1 mmHg), indicating respiratory acidosis. During OLV, pulse oximetry remained higher than 92%. The defect in the left main bronchus was successfully sutured after dissecting the fistula between the left main bronchus and the gastric conduit, and subsequently, OLV resulted in ideal ventilation.

**Lessons:**

A left-sided GBF could lead to leakage from the OLV during surgery. Possible aspiration or alveolar hypoventilation due to this leakage is a major concern during airway management before surgical repair of the main bronchus.

## 1 Introduction

The most common surgical intervention for resectable esophageal carcinoma is esophagectomy with gastric conduit reconstruction.^[1]^ A gastrobronchial fistula (GBF) between the main bronchus and the gastric conduit is a rare and complex post-surgical complication. The most likely cause of the fistula is an esophageal leak from the surgical anastomosis, which invades the posterior wall of the tracheobronchial tree.^[2]^ GBFs are also a potential complication of radiation therapy. The individual case reports over the past few decades describe only a few invasive and noninvasive treatments. Even though surgical interventions are often highly invasive and involve the interposition of omental, pleural, or muscle flaps, direct closure of both bronchial and esophageal defects is preferred.^[3]^ Mild GBF is typically managed with less-invasive interventions, such as esophageal or tracheobronchial stenting, fibrin glue, and over-the-scope clipping.^[4–7]^ Additionally, conservative management strategies have also been described and are employed when the patient is clinically stable and if the fistula is not associated with mediastinitis or other pulmonary complications.^[8]^ However, there are no standard treatment strategies for this rare condition,^[9]^ and airway management during anesthesia for surgery is both important and challenging.

## 2 Methods

We describe our strategy for airway management during general anesthesia in a patient with GBF who underwent left main bronchus fistula closure and gastric conduit repair with an intercostal flap. The patient provided written informed consent for the publication of this case report.

## 3 Case presentation

**Figure 1:**
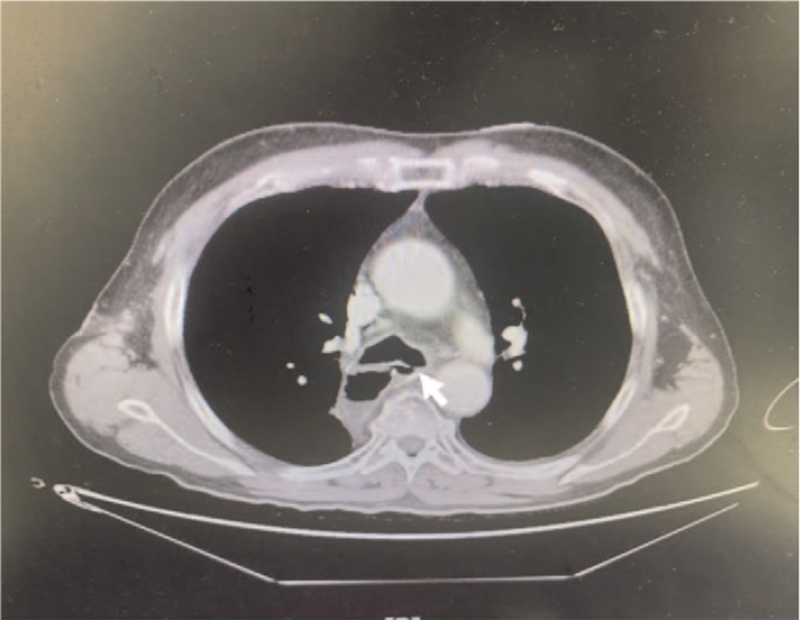
Computed tomography of the chest shows a fistula between the left main bronchus and the gastric conduit, indicated by a white arrow at the level of the carina.

**Figure 2:**
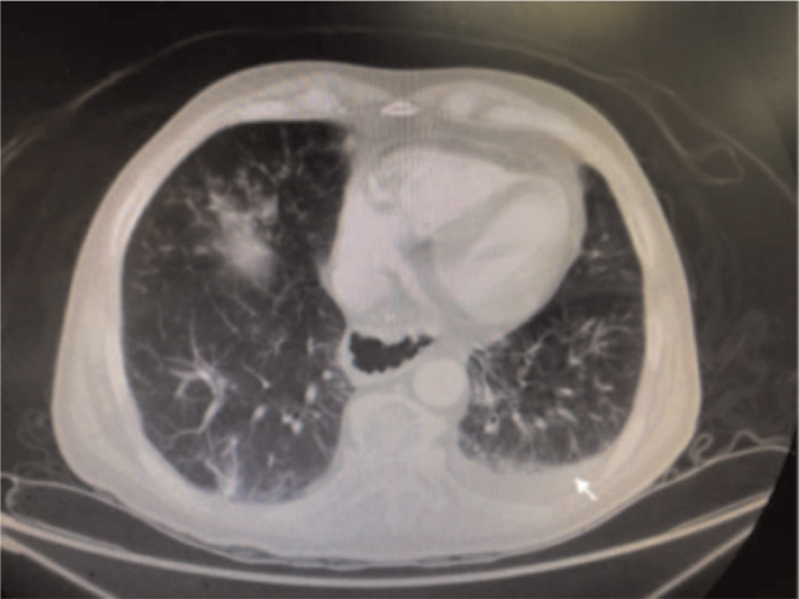
Computed tomography of the chest shows infiltration of the left lower lobe with pleural effusion, indicated by white arrow.

**Figure 3:**
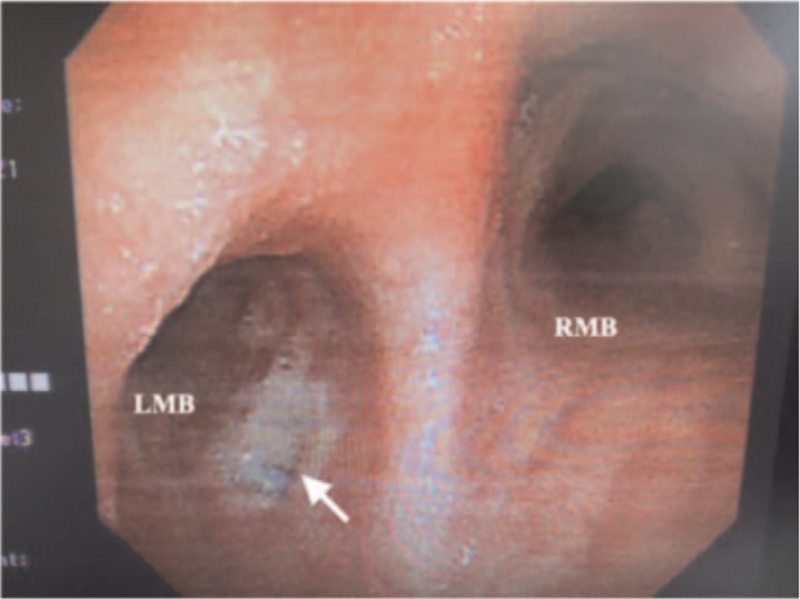
View of the carina under bronchoscopy, the fistula orifice is shown by a white arrow. LMB = left main bronchus, RMB = right main bronchus.

A 63-year-old man who had undergone neoadjuvant concurrent chemoradiotherapy for esophageal squamous cell carcinoma (cT3N2M0) required thoracoabdominal esophagectomy with reconstruction by gastric pull-up and tabularization of the gastric conduit (Ivor-Lewis procedure) in April 2021. About 8 weeks later, he experienced repeated fevers for a week, accompanied by a cough with copious sputum, dysphagia, and chest tightness. A chest x-ray indicated aspiration pneumonia, and he was hospitalized for antibiotic treatment. A gastroscopy showed no stenosis along the alimentary tract, but there was an air leak at the surgical anastomosis. Furthermore, a thoracic computed tomography revealed infiltration in the left lower lung with pleural effusion, along with a suspected left-sided GBF located 2 cm below the carina (Figs. 1 and 2). Bronchoscopy confirmed the GBF (Fig. 3), and surgery, that is, gastric conduit repair with intercostal flap and fistula closure in the left main bronchus, was recommended.

### 3.1 Preoperative anesthesia evaluation

The patient underwent a preoperative anesthesia evaluation during treatment with broad-spectrum antibiotics for aspiration pneumonia. His history included chronic smoking, a daily habit of drinking and chewing betel nuts, apart from a lower-third esophageal squamous cell carcinoma treated with neoadjuvant concurrent chemoradiotherapy and the Ivor-Lewis procedure. However, no relevant family medical history was present. At the evaluation, he weighed 60 kg and his vital signs were blood pressure 117/75 mmHg, heart rate 96 beats/min, respiratory rate 16/min, and pulse oximetry 89% saturation on room air. Blood tests and laboratory evaluations were remarkable for low hemoglobin (9.0 g/dL; normal, 13.7–17 g/dL), high alanine aminotransferase (49 IU/L; normal, 5–40 IU/L), and low albumin (2.8 g/dL; normal, 3.8–5.3 g/dL). Auscultation revealed bilateral rales, and his airway was evaluated as Mallampati class II with a thyromental distance of >6 cm with an inter-incisor gap of 5 cm upon full neck extension and lower incisor shaking. The patient provided informed consent for surgery and anesthesia before the procedure.

### 3.2 Anesthesia management and surgical experience

Based on preoperative anesthesia evaluation, we planned to use general anesthesia with OLV. After preoperative preparation, the patient received intravenous fentanyl.2μg/kg, propofol 2 mg/kg mixed with lidocaine 20 mg, and cisatracurium 0.5 mg/kg for conventional induction. The radial artery in the left hand was catheterized for invasive blood pressure monitoring. Another peripheral 18-gage venous catheter was placed in the forearm for intraoperative blood transfusion, and a nasogastric tube was inserted for decompression of the gastric conduit. Propofol and fentanyl were infused for intraoperative anesthesia maintenance using a target-controlled infusion system. The depth of anesthesia was monitored by the bispectral index (BIS) value, which was maintained between 40 and 60 for both anesthesia induction and surgery.

A right-sided, double-lumen endotracheal tube was used for intubation. A left-sided OLV with total intravenous anesthesia (TIVA) was used instead of inhalational anesthetics. Under these conditions, a PEEP of 6 cmH_2_O and nasogastric tube decompression can generate positive pressure across the fistula would prevent backflow into the left bronchus. The absence of backflow was verified by intraoperative fiberoptic bronchoscopy. However, as the anesthesia apparatus indicated a circuit leak, the oxygen flow rate increased to 10 L/min to temporarily solve this problem. Initial arterial blood gas analysis indicated respiratory acidosis as pH was 7.17, PaO_2_ was 84.2 mmHg at 100% oxygenation, and PaCO_2_ was 77.1 mmHg (hypercapnia). Nevertheless, pulse oximetry could be maintained at or above 92% with OLV. Resolving adhesions from previous thoracoscopic wounds and individual explorations of the pulmonary and gastric conduits, the right main bronchus, and the subcarinal and left main bronchus were time-consuming. The left main bronchus was successfully sutured after the fistula between the left main bronchus and the gastric conduit had been dissected. This repair not only led to ideal alveolar ventilation with OLV, but also the immediate correction of respiratory acidosis.

Subsequently, the surgeon turned an intercostal flap to cover the defect in the gastric tube by suturing the muscle flap to the pulmonary parenchyma and the gastric conduit. The surgery lasted approximately 13 hours, and the patient's intraoperative hemodynamic state was relatively stable except for a brief period of tachycardia at 113 beats/min. After surgery, a single lumen endotracheal tube was placed, and the patient was transferred to the intensive care unit for mechanical ventilation support and pneumonia continued to be treated with cefoperazone and teicoplanin. Six days later, the patient was extubated and transferred to an ordinary ward the next day. An esophagogram and bronchoscopy on postoperative day 11 showed no fistula. His nasogastric tube and chest tubes were removed on postoperative day 11 and 13. The patient's postoperative course was uneventful and without complications and he was discharged home after 3 weeks.

## 4 Discussion

### 4.1 Literature review

GBF might develop either in the early or the late postoperative period after esophagectomy with gastric pull-up, and can occur anywhere in the respiratory tract, from the trachea to the lobar bronchus. The most common clinical signs and symptoms are cough on swallowing, followed by fever, choking, chest pain, dyspnea, pneumonia, and life-threatening hemoptysis;^[10]^ respiratory failure with septic shock is the most common cause of death.^[11]^ A thoracic computed tomography scan with contrast, gastroscopy, bronchoscopy, and upper gastrointestinal contrast studies typically confirm GBF presence. As patients often present with malnutrition and repeated pneumonia, prompt administration of broad-spectrum antibiotics, gastric drainage, maintenance of fluid and electrolyte balance, and chest physiotherapy are necessary before definitive surgery. Even though there is no standard treatment for GBF, several interventions have been proposed in the past 20 years, such as esophageal stenting, tracheobronchial Y-shaped stenting, over-the-scope clips, fistula plugs, and surgical repair with muscle flaps or bovine pericardium.^[2,6,9,12–14]^ Interestingly, Li et al have demonstrated that surgical repair has a greater long-term survival rate than other non-invasive treatments.^[10]^

### 4.2 Anesthesia management

We used right-sided video-assisted thoracoscopic surgery for surgical repair with intercostal muscle flaps because a right-sided, double-lumen endotracheal tube is indicated for lesions in the left main bronchus. Air leak was our major concern during surgery since it would lead to hypercapnia, desaturation due to alveolar hypoventilation, and aspiration from backflow due to excessive gastric pressure. Therefore, PEEP and nasogastric tube decompression were used to generate positive pressure across the fistula to prevent backflow into the left bronchus during mechanical ventilation.

OLV is commonly employed to facilitate surgical access in the intra-thoracic cavity and hypoxemia during OLV has been reported to occur in approximately 5% of all thoracic surgeries.^[15]^ Therefore, propofol and fentanyl were infused using a target-controlled infusion system for intraoperative oxygen saturation maintenance and to guarantee minimal suppression of hypoxic pulmonary vasoconstriction (HPV).

### 4.3 HPV

HPV is the mechanism by which the lungs optimize blood oxygenation, that is, by avoiding V/Q mismatch. Specifically, when focal alveolar hypoxia occurs, HPV causes pulmonary vasoconstriction of the corresponding area, which preserves oxygenation by decreasing intrapulmonary shunting.^[16]^ Decreasing PaO_2_, the main stimulus for HPV, results in precapillary vasoconstriction and redistribution of pulmonary blood flow away from hypoxemic lung regions via a pathway involving nitric oxide and/or cyclooxygenase synthesis inhibition.^[17]^ Most anesthetic drugs have a negative effect on HPV, and all inhalational anesthetics, particularly and dose-dependently, inhibit HPV. In contrast, common intravenous anesthetic drugs, such as propofol, do not affect HPV.^[18]^

### 4.4 TIVA and BIS

TIVA was first described in 1872 by Pierre-Cyprien when he used chloral hydrate for anesthesia, and in the next century, thiopentone, benzodiazepines, and ketamine were introduced. Compared to inhalational anesthetics, TIVA's disadvantage is delayed emergence from anesthesia upon barbiturate or benzodiazepine use for long procedures. In contrast, propofol, introduced in 1977, is widely used, has a rapid recovery profile, and its action involves inhibition of the neurotransmitter γ-aminobutyric acid (GABA) function through the GABA_A_ receptor. It provides hypnosis, amnesia, and surgical immobility. It also minimizes postoperative side effects, especially emergence delirium, nausea, and vomiting.^[19,20]^ In our patient, TIVA was used to preserve HPV function and avoid adverse effects of inhalational anesthetics. Studies also have suggested that the BIS index, a computer-processed electroencephalography score that ranges from 0 (no electroencephalography activity) to 100 (fully awake), can predict responses to noxious stimulation during TIVA.^[21]^ BIS monitoring only requires the application of disposable electrodes on the forehead. Recent studies have demonstrated that BIS-guided anesthetic delivery of TIVA reduces propofol requirement, can better maintain desired depth of anesthesia and reduces recovery time.^[22]^

### 4.5 Hypercapnia

During surgery, arterial blood gas analysis revealed respiratory acidosis, and the hypercapnia could not be corrected until after the left main bronchus had been repaired. Several studies have suggested that hypercapnia, also known as permissive hypercapnia, has some advantages, such as reducing pulmonary inflammation, alveolar oxidative stress, and mortality in acute lung injury.^[23]^ However, it may also have detrimental effects, including decreased alveolar fluid clearance and impaired tissue repair. Although it is challenging to employ video-assisted thoracoscopic surgery for GBF repair, surgeons need to determine the surgical procedure needed for repairing the main bronchus at the earliest possible to avoid prolonged hypercapnia and alveolar hypoventilation. Prolonged hypercapnia has been shown to impair neutrophil function, contributing to greater lung injury and worse compliance.^[24]^

## 5 Conclusions

GBF is a rare and devastating complication after esophagectomy that needs immediate repair. Airway management in such procedures is always challenging and requires careful consideration to avoid additional complications.

## Acknowledgments

The authors thank the anonymous reviewers and the editor for their comments. We also thank Ms. Merissa, the editor of Enago (www.enago.tw), for her linguistic assistance during the preparation of this manuscript.

## Author contributions

**Conceptualization:** Sih-Yu Wang, En-Bo Wu.

**Data curation:** Wei-Chin Yuan.

**Supervision:** En-Bo Wu.

**Writing – original draft:** Sih-Yu Wang.

**Writing – review & editing:** En-Bo Wu.
